# Machine learning imaging applications in the differentiation of true tumour progression from treatment‐related effects in brain tumours: A systematic review and meta‐analysis

**DOI:** 10.1111/1754-9485.13436

**Published:** 2022-05-22

**Authors:** Abhishta Bhandari, Ravi Marwah, Justin Smith, Duy Nguyen, Asim Bhatti, Chee Peng Lim, Arian Lasocki

**Affiliations:** ^1^ Townsville University Hospital Townsville Queensland Australia; ^2^ College of Medicine and Dentistry James Cook University Townsville Queensland Australia; ^3^ Institute for Intelligent Systems Research and Innovation Deakin University Melbourne Victoria Australia; ^4^ Department of Cancer Imaging Peter MacCallum Cancer Centre Melbourne Victoria Australia; ^5^ Sir Peter MacCallum Department of Oncology The University of Melbourne Melbourne Victoria Australia

## Abstract

**Introduction:**

Chemotherapy and radiotherapy can produce treatment‐related effects, which may mimic tumour progression. Advances in Artificial Intelligence (AI) offer the potential to provide a more consistent approach of diagnosis with improved accuracy. The aim of this study was to determine the efficacy of machine learning models to differentiate treatment‐related effects (TRE), consisting of pseudoprogression (PsP) and radiation necrosis (RN), and true tumour progression (TTP).

**Methods:**

The systematic review was conducted in accordance with PRISMA‐DTA guidelines. Searches were performed on PubMed, Scopus, Embase, Medline (Ovid) and ProQuest databases. Quality was assessed according to the PROBAST and CLAIM criteria. There were 25 original full‐text journal articles eligible for inclusion.

**Results:**

For gliomas: PsP versus TTP (16 studies, highest AUC = 0.98), RN versus TTP (4 studies, highest AUC = 0.9988) and TRE versus TTP (3 studies, highest AUC = 0.94). For metastasis: RN vs. TTP (2 studies, highest AUC = 0.81). A meta‐analysis was performed on 9 studies in the gliomas PsP versus TTP group using STATA. The meta‐analysis reported a high sensitivity of 95.2% (95%CI: 86.6–98.4%) and specificity of 82.4% (95%CI: 67.0–91.6%).

**Conclusion:**

TRE can be distinguished from TTP with good performance using machine learning‐based imaging models. There remain issues with the quality of articles and the integration of models into clinical practice. Future studies should focus on the external validation of models and utilize standardized criteria such as CLAIM to allow for consistency in reporting.

## Introduction

Treatment of brain tumours with radiotherapy and/or chemotherapy often leads to the development of treatment‐related effects (TRE), including pseudoprogression (PsP) and radiation necrosis (RN), which can be difficult to distinguish from true tumour progression (TTP).^1^ This is a major diagnostic challenge with important clinical implications. For example, a successful treatment may be incorrectly ceased if PsP is not identified; conversely, there may be a delay in instituting the correct treatment if TTP is not diagnosed. PsP is particularly recognized as an issue in the management of glioblastomas,^2^ where standard of care includes maximal resection of the tumour and radiotherapy plus chemotherapy.^3^ A substantial proportion of patients receiving treatment for glioblastoma develop PsP, with the literature reporting an incidence of between 10 and 30%.^2^ Difficulties in distinguishing PsP from true tumour progression (TTP) are not exclusive to glioblastoma either, with an incidence of 21% also reported for low‐grade gliomas.^4^ PsP is radiologically defined as an increase in contrast uptake after the completion of radiotherapy without true tumour growth, which reduces or improves without an alteration of treatment.^5^ Clinical definitions of PsP vary significantly, with some studies utilizing a 6‐month follow‐up period for diagnosis and others using 2‐month follow‐up.^6^ RN is recognized as a separate entity that generally occurs at a later stage after treatment than PsP and is particularly considered a diagnostic challenge in brain metastases treated with stereotactic radiosurgery (SRS). The increased clinical manifestation of late TRE such as RN can be attributed to the improved median survival of patients due to treatment with SRS and improvements in systemic treatments (such as immunotherapy).^7^, ^8^ The literature reports the incidence of RN in patients treated with SRS for brain metastases as 24–26%.^9^, ^10^ RN most commonly occurs 6–24 months after completion of treatment,^11^ but can occur as early as 3 months to as late as 19 years after treatment.^12^

Follow‐up to determine whether the imaging changes regress is frequently impractical in the clinical setting; thus, there is a clinical need for making the distinction between TRE and TTP when routine imaging raises concern. Current imaging diagnostics to differentiate PsP from TTP in gliomas include the RANO (Response Assessment in Neuro‐Oncology) criteria^13^ and the more recent modified RANO criteria.^14^ However, an accuracy of only 82% has been reported using the RANO criteria to differentiate PsP from TTP,^12^ and assessment is subject to inter‐observer variability.^15^ Conventional MRI has limitations in the differentiation of TTP from RN in brain metastases treated with SRS.^7^, ^8^ The current gold standard for differentiating TRE from TTP remains histopathology, but there is the potential for patient morbidity due to its invasive nature.^2^ As a result, clinical decisions are often guided by imaging without histological confirmation.

These challenges have prompted research into computational methods, such as Artificial Intelligence (AI) methodologies, as an alternative non‐invasive point of care method of distinguishing between TRE and TTP, expecting to overcome the limitations of conventional imaging interpretation. Firstly, the learning and generalization capabilities of AI models for objective assessment offer a means to address the issue of inter‐observer variability. Secondly, AI models can identify subtle imaging features not noticeable and appreciable with the human eye, improving the ability to provide a ‘virtual biopsy’ indicative of tumour histological characteristics.^16^ In isolation, or in combination with human radiologist assessment, there is an expectation that the use of AI models would result in an increase in accuracy.

A simplified example of a typical ML pipeline is as follows: the image is (i) acquired through a standard clinical and/or dedicated research protocol; (ii) segmented, which can be conducted manually or semi‐automated/fully automated by algorithms performed on the whole brain; (iii) features are extracted, whether pre‐defined as in the case of *radiomics*, or without a pre‐definition in the case of *deep learning* (DL); (iv) features are selected as to remove redundant features and reduce computational power; (v) the ML *algorithm* is applied to a training data set, in which each data sample is linked to a desired clinical outcome (such as PSP); (vi) the trained ML *model* is applied to a previously unseen *test* data set for verification; (vii) a receiver operator (ROC) or precision recall curve is generated and the performance is measured, typically through the area under the curve (AUC) and the associated sensitivity and specificity scores.

Given the potential advantages of ML models, the aim of this study was to perform a diagnostic test accuracy (DTA) systematic review examining the existing ML models for differentiating between TTP and TRE in brain tumours, examining gliomas and metastases separately. A diagnostic test accuracy (DTA) meta‐analysis is also performed.

## Methods

The methods followed were in line with the PRISMA‐DTA (Preferred Reporting Items for a Systematic Review and Meta‐analysis of Diagnostic Test Accuracy Studies) guidelines.

### Search strategy

The search was performed on 22 September 2020 in the following databases: PubMed, Scopus, Embase, Medline (Ovid) and ProQuest. The search was subsequently updated on 10 January 2021. The search strategy for PubMed is demonstrated below, with appropriate adaptations made as required in each database.

(‘Machine learning’ OR ‘Neural network’ OR ‘Deep learning’ OR ‘Artificial intelligence’ OR ‘AI’ OR radiomic*) AND (glioma OR glioblastoma OR ‘brain tumor’ OR ‘brain tumor’ OR ‘brain neoplasm’ OR ‘brain cancer’) AND (pseudoprogression OR progression OR recurrence OR ‘radiation necrosis’).

### Inclusion and exclusion criteria

Studies were included if they met the following criteria: (i) Differentiated either glioma or metastasis TRE (RN and/or PsP) using computational imaging features, pre‐defined (such as radiomics) or DL‐derived; (ii) involved treatment with radiotherapy and/or chemotherapy; (iii) included adequate information that met the ML processing pipeline requirements, such as imaging acquisition parameters, segmentation method, features used, ML models and classification of results through follow‐up or histopathology; (iv) reported an AUC from a ROC curve or precision recall curve. The associated confidence interval or standard error was reported where available. If the ML processing pipeline characteristics were changed between experimentation, only the highest value was used. Only the test results (or validation) from ML models were used. Studies were excluded if they were non‐peer reviewed journal articles, reviews, studies focussing on paediatric patients, opinion articles and non‐English language articles.

### Data extraction and analysis

Data was extracted by the authors A.P.B and R.M, including the ML pipeline components and the main findings from the studies. The ML pipeline components extracted included the total number of patients in each group (whether TRE or TTP), imaging sequences used, segmentation method, features (as per PyRadiomics manual)^17^ and selection of ML models. The main findings included WHO grade, result (presented as an AUC, sensitivity and/or specificity) and diagnosis method. A meta‐analysis was performed in the STATAIC 12.0 software for groups that had sufficient data—the metandi package required a minimum of 4 studies with a reported sensitivity/specificity measure for the meta‐analysis. Pooled sensitivities and specificities were determined by back‐calculating and using hierarchical logistic regression.^18^ A hierarchical receiver operator curve (HSROC) was also generated for ML tasks that had more than four studies with appropriate data. A meta‐regression was performed in Meta‐DiSc^19^ on the meta‐analysed studies using a generalization of the Littenberg and Moses Linear model, which was weighted by the inverse of the variance.^20^ Co‐variates included non‐conventional imaging and deep learning use. A diagnostic odds ratio (dOR) and p‐value (where p < 0.05 indicated an effect) were reported. STATAIC was also used to generate a Deeks' funnel plot used for assessing publication bias in diagnostic test accuracy studies; this included a p‐value.^21^

### Quality assessment

The quality assessment of the studies was conducted based on the PROBAST (Prediction model Risk Of Bias ASsessment Tool), which is used to assess diagnostic test risk of bias and applicability.^22^ The Checklist for Artificial Intelligence in Medical Imaging (CLAIM)^23^ was also administered, as this is a ML‐specific checklist that may report further ML methodological/reporting deficiencies. It is a recently published 42‐item checklist and is part of the EQUATOR (Enhancing the QUAlity and Transparency Of health Research) Network guidelines specifically designed to improve the quality of studies for clinical uptake. Assessors collectively evaluated one article first to resolve differences in interpretation. For each study, items were then scored and deficiencies in sections were noted for discussion.

## Results

As demonstrated in the PRISMA flow diagram (see Fig. A2 in Appendix A), 1,081 papers were identified in the initial search, with 650 remaining after the removal of duplicates. After title and abstract screening, 65 articles were selected for full‐text analysis. There were 21 articles deemed eligible for inclusion—a total of 28 articles were excluded as they were conference abstracts, 2 did not employ ML, 12 did not compare TRE and TTP using ML models, and 2 did not separate primary tumours and metastases. Four additional articles^24^, ^25^, ^26^, ^27^ were found from an update of the literature search on 10/01/2021, resulting in a total of 25 articles.

**Fig. 1:**
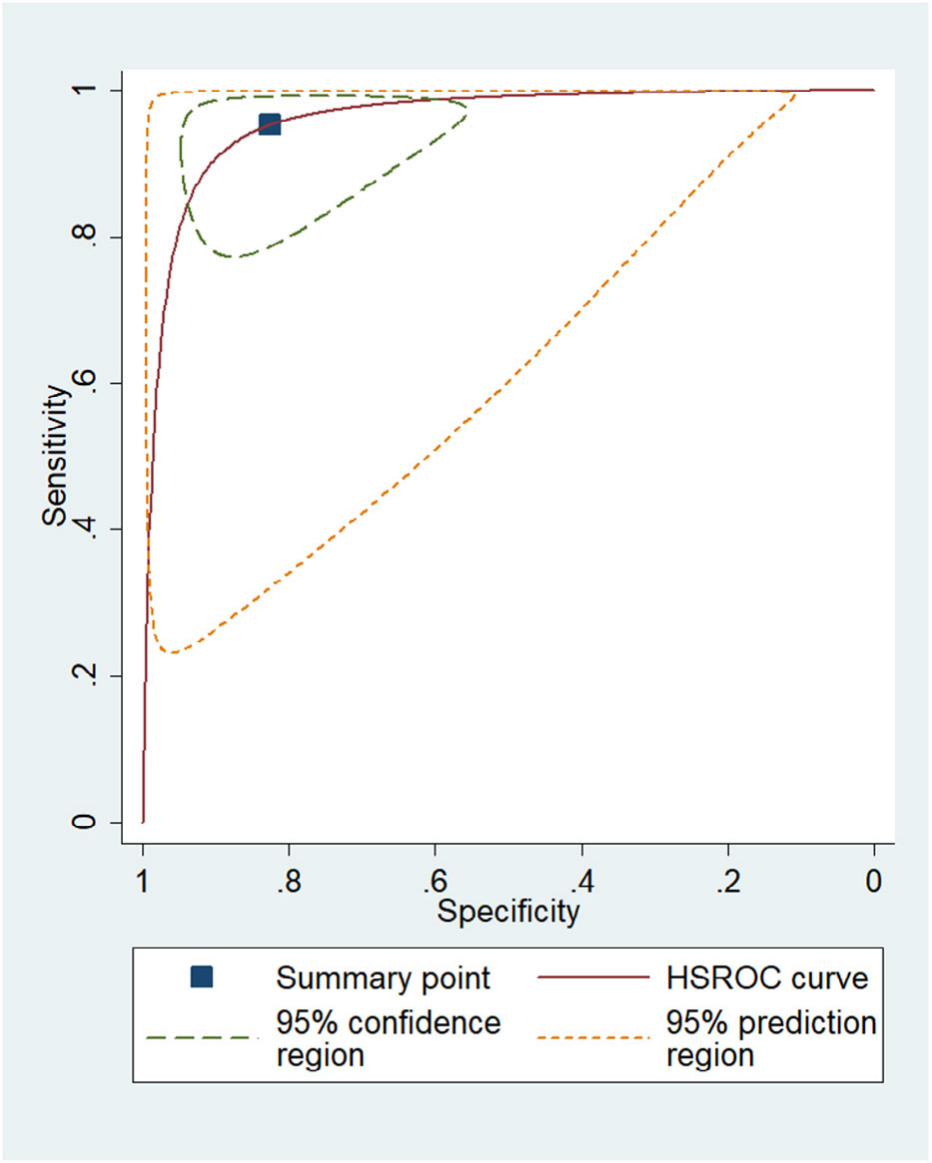
Hierarchical summary receiver operator curve for PsP versus TTP in gliomas (All Grades).^27^, ^28^, ^29^, ^30^, ^31^, ^32^, ^33^, ^34^, ^35^ [Colour figure can be viewed at wileyonlinelibrary.com]

Findings were reported as: ML pipeline components, clinical findings and main results (Table A1 in Appendix A). The results were then divided into the following four categories:

1 *Gliomas (all grades), PsP vs TTP:* The highest performing pipeline for gliomas (all grades) PsP vs. TTP was AUC = 0.98, accuracy of 88.02%, sensitivity of 99.24% with a specificity of 66.04% using DL‐derived features and a convolutional neural network (CNN) model on DTI.^28^ The range of AUC = 0.81–0.98, accuracy = 70–95.6%, sensitivity = 80–100%, specificity = 40–97.93% in 16 studies. A meta‐analysis was performed on 9 studies^27^, ^28^, ^29^, ^30^, ^31^, ^32^, ^33^, ^34^, ^35^ which had sufficient outcomes (reported sensitivity and specificity). A sensitivity of 95.2% (95%CI: 86.6–98.4%) and specificity of 82.4% (95%CI: 67.0–91.6%) were found. The HSROC is displayed in Fig. 1. The meta‐regression showed that studies incorporating advanced MRI sequences (conventional sequences defined as T1, T2, FLAIR, DWI and ADC) provided superior performance (dOR = 6.55 [95%CI = 1.29–33.27] *P* = 0.0291) and that DL models were not superior to classical ML models (dOR = 6.36 [95%CI = 0.41–98.44] *P* = 0.1545) although limited by a large effect size.
2 *Gliomas, RN vs TTP:* The highest performance was AUC = 0.9988, sensitivity = 99.07% and specificity = 97.93% using handcrafted and DL‐derived features on conventional MRI sequences.^36^ The reported range of AUC was between 0.891 and 0.998, accuracy between 79.2% and 83.79%, sensitivity between 75% and 99.07% and specificity 79% to 97.93% in 4 studies.
3 *Gliomas, TRE (includes both PsP and RN) vs TTP:* The highest performance was AUC = 0.94 (95%CI = 0.7788–1.0000), accuracy of 93.33%, sensitivity of 100% and specificity of 90% using radiomic features and an SVM (support vector machine) model on conventional MRI sequences.^1^ The reported AUC ranged from 0.80 to 0.94, accuracy between 82% and 93.33%, sensitivity between 98.31% and 100% and specificity between 60% and 96.97% in 3 studies.
4 *Metastasis, RN vs TTP:* The highest scoring pipeline achieved AUC = 0.81, sensitivity = 65.38% and specificity = 86.67% using shape and texture features with an SVM model on conventional MRI sequences.^7^ For the second study, an AUC of 0.73 was reported with an accuracy of 73.2%.

### Quality assessment and risk of bias

**Fig. 2:**
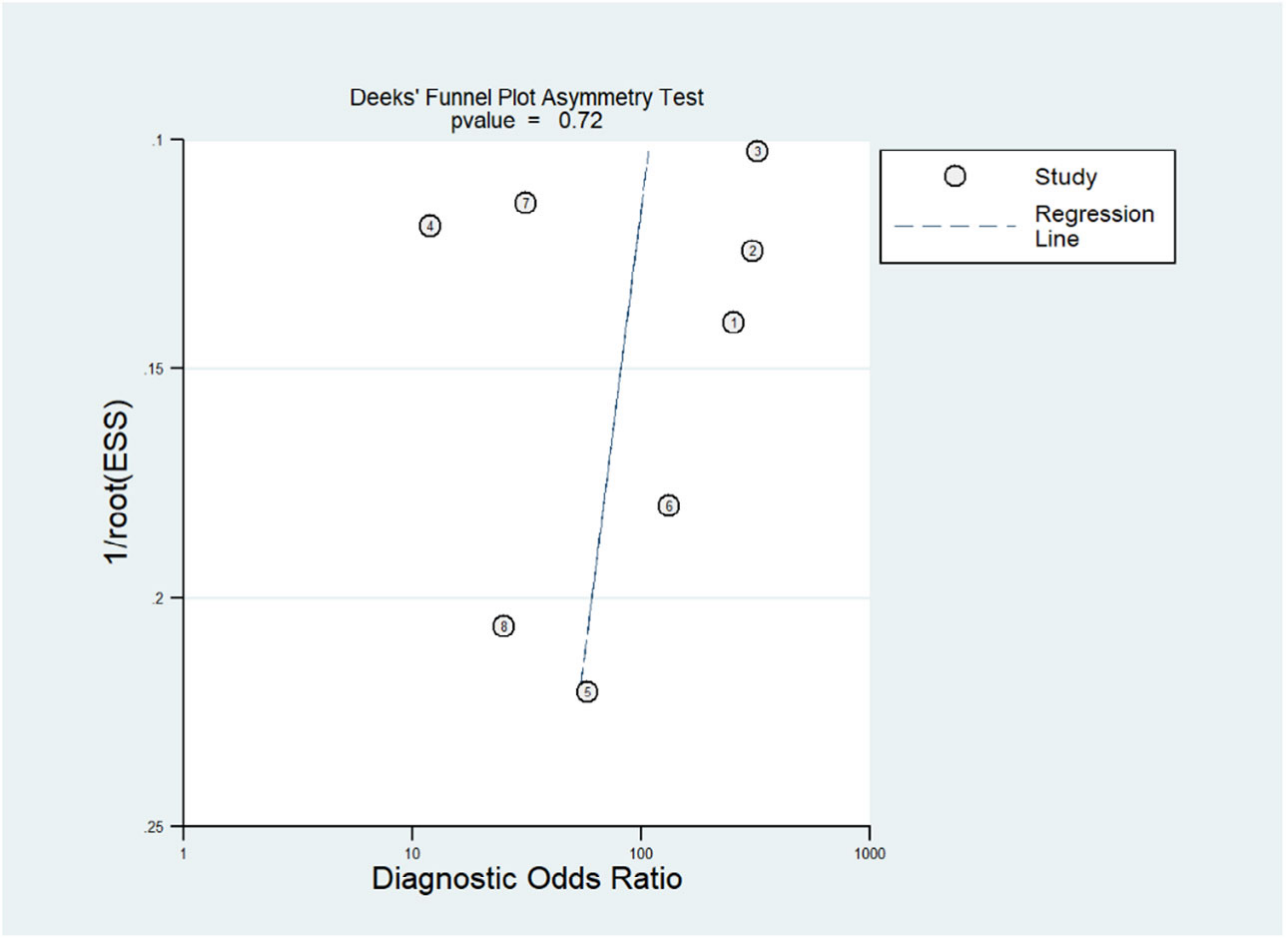
Deeks' funnel plot for PsP versus TTP in gliomas (All Grades).^27^, ^28^, ^29^, ^30^, ^31^, ^32^, ^33^, ^34^, ^35^ [Colour figure can be viewed at wileyonlinelibrary.com]

**Fig. A1:**
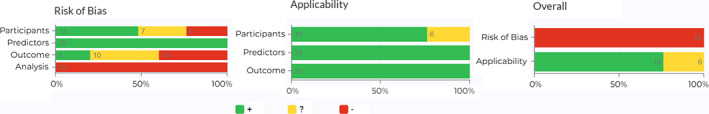
PROBAST quality scoring of included studies.

**Fig. A2:**
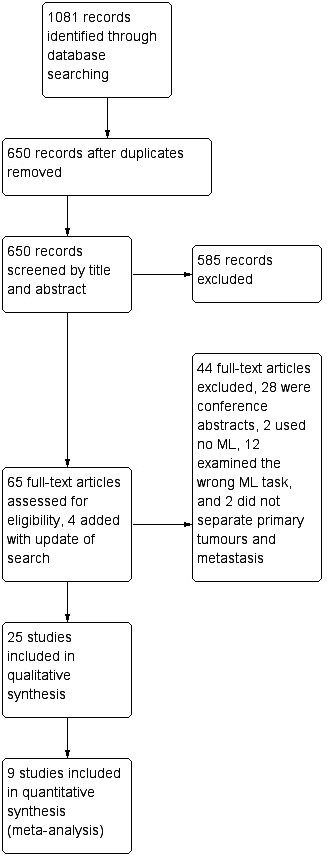
Flow diagram of included studies.

The quality assessment using the CLAIM criteria demonstrated an overall mean score across all studies of 19.4 out of 42 (range 10–30, standard deviation = 5.19). The Appendix A (Fig. A1 and Table A2) demonstrates the results from CLAIM quality assessment and the PROBAST assessment. Risk of bias assessment using PROBAST demonstrated an overall high risk of bias in all studies. Conversely, applicability was high (76%) within included studies. The Deeks' funnel plot (Fig. 2) showed a low publication bias (*P* = 0.72) for the 9 studies^27^, ^28^, ^29^, ^30^, ^31^, ^32^, ^33^, ^34^, ^35^ appropriate for the meta‐analysis (those comparing PsP versus TTP in gliomas of all grades).

## Discussion

In summary, there were three tasks on gliomas (PsP vs. TTP, RN vs. TTP and TRE vs. TTP) and one task on metastases (RN vs. TTP). Twenty‐five articles assessed the four ML tasks, with AUCs ranging from 0.73^8^ to 0.98.^28^ The majority (16 studies) investigated the differentiation between PsP and TTP in gliomas, with only 2–4 studies considering the other three ML tasks. A meta‐analysis was able to be performed on the first task: PsP vs. TTP in gliomas. Pooled sensitivity of 95.2% (95%CI: 86.6–98.4%) and pooled specificity of 82.4% (95%CI: 67.0–91.6%) were found upon combining the 9 studies.^27^, ^28^, ^29^, ^30^, ^31^, ^32^, ^33^, ^34^, ^35^ The wide confidence intervals for the meta‐analysis, especially for the specificity, are notable; these are attributed, at least in part, to heterogeneity within pipelines. In addition, the HSROC demonstrates a large prediction region. In the same 9 studies,^27^, ^28^, ^29^, ^30^, ^31^, ^32^, ^33^, ^34^, ^35^ advanced sequences showed higher diagnostic potential than conventional sequences. DL models were not superior to classical ML models, although there were large effect sizes. This systematic review found that studies utilizing histopathological diagnosis of gliomas as the ground truth had accuracies of 90.82%^34^ and 87.3%.^29^ These accuracies are higher than the accuracy of the RANO criteria, which has been reported in the literature as 82%.^37^

For ML applications in gliomas comparing PsP and TTP, the highest AUC was found using DTI and a CNN model (AUC = 0.98).^29^ This was much higher than using conventional MRI sequences (AUC = 0.85),^30^ which increased to AUC = 0.96 when integrating diffusion/perfusion imaging in one study.^34^ Utilization of FET‐PET revealed comparable performance (AUC = 0.93).^25^ Comparing RN and TTP in gliomas, the highest performing pipeline used conventional MRI sequences (T1 and T2‐FLAIR) (AUC = 0.94), using SVM.^24^ The use of MET‐PET and FDG‐PET imaging did not increase performance (AUC = 0.891).^38^ Metastasis ML applications comparing RN and TTP demonstrated the lowest maximum AUC = 0.81 using just T1‐Gd and FLAIR and SVM.^7^ These results demonstrate varying performances of imaging sequences, imaging modalities and ML models based on the specific ML tasks.

Whilst the results of the studies thus far are promising, the quality assessment process demonstrated a number of deficiencies within the primary studies that may influence the generalizability and clinical applicability of these results. For example, a number of studies had small sample sizes (*n* < 100) and only 4 studies incorporated external validation. External validation remains a key issue, as ML models need to be validated on multi‐institutional cohorts for translatability. There was also a lack of failure analysis and inadequate reporting of performance statistics for these models. Many studies also lacked clear inclusion and exclusion criteria, whilst others did not accurately define the time periods for PsP and RN. Furthermore, numerous studies relied on expert opinion, self‐made definitions and the RANO criteria as the ground truth reference for the differentiation of TTP and TRE, rather than histopathology, despite the inaccuracies of these methods.

Our review has a number of limitations that affect the strength of the analysis. There was a small number of studies in three of the four categories investigated. Furthermore, there was substantial heterogeneity between studies, with studies varying in the imaging modalities used, ML methods and tumour grades. There was also a lack of consistency in the definitions of PsP and RN. This highlights the lack of consensus surrounding the reporting of the methodology and results in AI studies in medical imaging. The application of CLAIM for further studies will prove valuable for the consistency in reporting required for the comparative analysis of ML papers. Additionally, articles that combined metastasis and gliomas were excluded, which limited the number of studies available for meta‐analysis; however, this was necessary to reflect clinical practice more accurately.

Future research looks promising for the integration of AI methodologies into clinical practice. Efforts are already being made by the development of guidelines such as the CONSORT‐AI (Consolidated Standards of Reporting Trials—Artificial Intelligence) extension which aims to streamline the reporting of clinical trials involving AI.^39^ A similar guideline includes the SPIRIT‐AI (Standard Protocol Items: Recommendations for Interventional Trials–Artificial Intelligence) extension for clinical trial protocols.^40^ Guidelines such as the TRIPOD‐AI (Transparent Reporting of a multivariable prediction model for Individual Prognosis Or Diagnosis‐Artificial Intelligence)^41^ and QUADAS‐AI (Quality Assessment of Diagnostic Accuracy Studies‐Artificial Intelligence)^42^ extension are yet to be released.

In conclusion, this systematic review and meta‐analysis demonstrated high sensitivity and specificity for ML imaging applications in the differentiation of TTP from TRE in brain tumours, especially in gliomas. Despite promising findings, there remain issues with heterogeneous methodology, the quality of available studies, as well as the clinical integration of models. Future higher quality, prospective studies are required to further investigate the role of ML in the differentiation of TTP from TRE and how this can be incorporated into routine clinical practice.

## Funding

Arian Lasocki: *Grant*: Peter MacCallum Cancer Foundation, *Comments*: Arian Lasocki was supported by a Peter MacCallum Cancer Foundation Discovery Partner Fellowship.

## Acknowledgements

Open access publishing facilitated by James Cook University, as part of the Wiley ‐ James Cook University agreement via the Council of Australian University Librarians.

## Data availability

The data supporting the findings from the study are available from the corresponding author upon reasonable request.

## Ethical approval

No ethics was required as this was a review of existing evidence.

## Appendix A

**Table A1:** Pipeline features in the differentiation of TRE and TTP using machine learning

First Author, Year	WHO Grade	Number	Imaging sequences	Segmentation	Features and selection	Machine Learning Method	Result	Confirmation
Glioma (All Grades): PsP versus TTP									
Jang 2020^43^	Glioblastoma (IV)	PsP = 38 TTP = 66	T1‐Gd	Unclear	CNN‐LSTM learning derived features	CNN	AUC = 0.86	Based on expert opinions
Liu 2020^28^	Glioblastoma (IV)	PsP = 23 TTP = 61	DTI	Pipeline performed on whole brain	DL features Human‐selected	CNN	DenseNet produced an AUC = 0.98, accuracy of 88.02%, sensitivity of 99.24% with a specificity of 66.04%	Final diagnosis made from follow‐up image interpretation and physician evaluation
Li 2020^44^	Glioblastoma (IV)	PsP = 23 TTP = 61	DTI	Pipeline performed on whole brain	DL features using deep convolutional generative adversarial network (DCGAN) and AlexNet NA	SVM	Using the last 2 convolutional layers from AlexNet the accuracy= 92%, sensitivity = 97.6%, specificity = 88.3%	Follow‐up images and professional evaluation by experienced clinicians
Kim 2019^34^	Glioblastoma (IV)	PsP = 46 TTP = 49	T1 T1‐Gd FLAIR DWI: ADC DSC: CBV	Semi‐automated with segmentation threshold and region‐growing segmentation	Shape Intensity Texture Wavelet‐ transformed LASSO logistic regression model	Generalized linear model	AUC of 0.96 (95% CI: 0.88–1.00), accuracy of 95.6%, sensitivity of 93.7% and specificity of 100%.	Final diagnosis of PsP made with increasing contrast‐enhancing lesions on MRI that subsequently regressed or became stable without any changes in the treatment for at least 6 months after therapy. Final diagnosis of TTP made if enhancing lesions gradually increased on more than 2 subsequent follow‐up MRI studies performed at 2‐ to 3‐month intervals and required a prompt change in treatment.
Elshafeey 2019^32^	Glioblastoma (IV)	PsP = 22 TTP = 83	DCE: K^trans^ DSC: rCBV	Semi‐automated	Histogram features Haralick Maximum Relevance Minimum Redundancy (MRMR)	SVM	Using SVM model a AUC = 0.89, accuracy = 90.82%, sensitivity = 91.4% and specificity = 88.2%	Histopathological tissue evaluation
Bani‐Sadr 2019^30^	Glioblastoma (IV)	PsP = 23 TTP = 53	T1 T1‐Gd FLAIR	Manual	Radiomic features Wilcoxon‐test–based method	Random forest	Radiomics and MGMT promoter status discriminated with an AUC = 0.85, accuracy = 79.2%, sensitivity = 80.0% (95% CI [56.3–94.3%]), and specificity= 75.0% (95% CI [19.4–99.3%])	Histopathological tissue evaluation or radioclinical follow‐up by RANO criteria
Jang 2018^45^	Glioblastoma (IV)	PsP = 30 TTP = 48	T1 T1‐Gd	Pipeline performed on whole brain	CNN‐LSTM DL structure and clinical features NA	CNN‐LSTM structure	An AUC = 0.83 was found using the model	CE lesion on follow‐up MRI based on RANO, Histopathological tissue evaluation or significant uptake on PET scans
Ismail 2018^46^	Glioblastoma (IV)	PsP = 71 TTP = 34	T2 FLAIR	Manual	Shape Local hand‐crafted features Sequential feed‐forward feature selection	SVM	An accuracy = 90.2% was found	Histologic analysis or follow‐up imaging
Booth 2017^31^	Glioblastoma (IV)	PsP = 9 TTP = 15	T2	Manual	Shape Intensity Minkowski functionals t‐tests	SVM	Accuracy = 86%, Sensitivity = 100% (95%CI: 51–100%), Specificity = 67% (95%CI: 21–94%)	RANO criteria Increasing enhancing lesion at 4 weeks, 4 months or 7 months following chemoradiotherapy
Zhang 2016^47^	Glioblastoma (IV)	PsP = 23 TTP = 56	DTI	Pipeline performed on whole brain	Deep (dictionary) learning Index‐based approach	SVM	An AUC = 0.87 was found using the model	On follow‐up by physicians clinical and imaging experience. Biopsy performed if necessary.
Qian 2016^48^	Glioblastoma (IV)	PsP = 13 TTP = 22	DTI	Pipeline performed on whole brain	Deep (dictionary) learning 28 top‐ranked features	SVM	Using locally linear embedding to extract 4 dimensions from the image an AUC = 0.875 (SD = 0.276) and accuracy of 77.0% (SD = 19%) was found	Follow‐up imaging with clinician experience or histopathological evaluation
Hu 2011^33^	Glioblastoma (IV)	PsP = 16 TTP = 15	T1 T2 FLAIR TTP DWI: ADC DSC: rCBF, rCBV, MTT	Pipeline performed on whole brain	Intensity NA	One‐Class SVM	AUROC= 0.9439, sensitivity = 89.91%, specificity= 93.72%	Follow‐up MRI scans acquired every 2–3 months following chemoradiotherapy
Akbari 2020^29^	Glioblastoma (IV)	PsP = 20 TTP = 63	T1 T1‐Gd T2 T2‐FLAIR DTI DSC	Manual	Intensity Shape Texture: GLCM, GLRLM Sequential feature selection	SVM	Radiomics produced the highest AUC = 0.919, accuracy of 87.3%, sensitivity of 80% with a specificity of 88.69%	Histopathological tissue evaluation
Lohmann 2020^27^	Glioblastoma: *n* = 33, Anaplastic astrocytoma (III): *n* = 1	PsP = 16 TTP = 18	FET‐PET	Three different segmentations based on tumor brain ratio of the SUV	Shape, intensity and texture: GLSZM SVM‐RFE	Random Forest	AUC = 0.73 Accuracy = 70% Sensitivity = 100% Specificity = 40%	Histopathological tissue evaluation or clinicoradiolocal by RANO follow‐up
Lee 2020^26^	Glioblastoma (IV) = 23; anaplastic astrocytoma (III) = 2; Grade II = 18;	PsP = 36 TTP = 7	T1 T1‐Gd T2 T2‐FLAIR DWI: ADC T1‐post–T1 minus pre‐contrast T2 minus FLAIR	Pipeline performed on whole brain	CNN‐LTSM deep learning model	CNN	AUC = 0.81 (95%CI: 0.73–0.87)	Histopathological tissue evaluation
Kebir 2020^25^	Glioblastoma (IV)	PsP = 14 TTP = 30	FET‐PET	NA	Handcrafted features	Linear discriminant analysis	AUC = 0.93 (95% CI: 0.78–1, sensitivity 100% specificity 80%)	Confirmatory MRI 4 weeks later
Gliomas (all grades): RN versus TTP									
Prasanna 2016^49^	Glioblastoma (IV)	RN= 24 TTP= 18	T1	Manual	CoLlAGe features (Co‐occurrence of Local Anisotropic Gradient Orientations) NA	Random Forest	Accuracy= 83.79 ± 5.43%	Histopathological tissue evaluation
Zhang 2019^36^	High Grade Gliomas (III/IV)‐ 32 Low Grade Gliomas (I/II)‐ 19	TTP = 35 RN = 16	T1 T1‐Gd T2 FLAIR	Manual segmentation	Handcrafted Features plus Inception v3 (deep learning derived features)	Logistic regression	Fusion Inception v3 deep learning achieved an AUC= 0.9988, Sensitivity = 99.07% Specificity = 97.93%	Histopathological confirmation or confirmed by imaging and clinical follow‐up by neuroradiologists (follow‐up time > 6 months)
Wang 2020^38^	Grade II Glioma = 72 Grade III Glioma = 45 Grade IV Glioma = 43	TTP = 118 RN = 42	FDG‐PET MET‐PET T1 FLAIR T1‐Gd	Manual segmentation	Texture LASSO	Logistic regression	FDG‐PET + MET PET: AUC = 0.891 (95% CI: 0.823–0.958) Accuracy = 79.2% Sensitivity = 75.0% Specificity = 91.7%	Clinicoradiological follow‐up MRI – minimum of 3 months
Gao 2020a^24^	Grade II = 41 Grade III = 32 Grade IV = 56 Unknown = 17	TTP = 96 RN = 50	T1 T2 T1‐Gd	Pipeline performed on whole brain	Deep learning derived features from ERN‐Net (efficient radionecrosis neural network)	Deep neural network	AUC (95% CI) = 0.92 (0.90–0.93) Accuracy (95% CI) = 81% (79–83%) Sensitivity (95% CI) = 82% (79–84%) Specificity (95% CI) = 79 (75–82%)	Histopathological tissue evaluation
Gliomas (all grades): TRE (RN and PsP) versus TTP									
Gao 2020b^1^	Anaplastic astrocytoma (III) = 7 Glioblastoma (IV) = 32	TRE = 14 TTP = 25	T1 T2‐FLAIR	Manual	Shape Intensity Texture: GLCM, GLSZM, GLRLM, NGTDM, GLDM RFE	SVM	A combination of both subtractions gave an AUC = 0.94 (95%CI = 0.7788–1.0000), accuracy of 93.33%, sensitivity of 100% and specificity of 90%	Histopathological tissue evaluation
Bacchi 2019^50^	High grade gliomas (3 Grade III, 52 Grade IV)	TRE = 16 TTP = 39	T1‐Gd FLAIR DWI: ADC	Pipeline performed on whole brain	CNN DL features NA	CNN	The highest AUC = 0.80 for DWI + FLAIR images; Accuracy = 82%, Sensitivity = 100%, Specificity = 60%	Histopathological tissue evaluation or on follow‐up MRI at >6 months.
Artzi 2016^51^	Glioblastoma (IV) = 18 Anaplastic Astrocytoma (III)= 2	TRE = 3 TTP = 12 Both = 1	DCE: K^Trans^, v_e_, k_ep_, v_p,_ Bolus arrival time (BAT)	Semi‐automated	K^Trans^, v_e_, k_ep_, v_p_ ANOVA	SVM	Training Data sens = 98.31% spec = 96.97%	Follow‐up at 2–3 months or histopathology
Metastasis: RN versus TTP									
Peng 2018^7^	Metastasis	TTP = 52 RN = 30	T1‐Gd FLAIR	Semi‐automatic using deep learning	Shape Texture	IsoSVM	AUC = 0.81 Sensitivity = 65.38% Specificity = 86.67%	Based on neuroradiologist interpretation
Zhang 2018^8^	Metastasis	TTP = 73 RN = 24	T1 T1‐Gd T2 FLAIR	Semi‐automated	Texture Concordance correlation coefficients	RUSBoost	AUC = 0.73 Accuracy = 73.2%	Histopathological resection or imaging on follow‐up

ADC, apparent diffusion coefficient; AUC, area under the curve; CBF, cerebral blood flow; CBV, cerebral blood volume; CNN, convolution neural network; DCE, dynamic contrast‐enhanced; DNN, deep neural network; DSC, dynamic susceptibility contrast; DTI, diffusion tensor imaging; DWI, diffusion weighted imaging; FDG‐PET, fluorodeoxyglucose (FDG)‐positron emission tomography; FET‐PET, O‐(2‐ [18F]fluoroethyl)‐L‐tyrosine (18F‐FET) positron emission tomography; FLAIR, fluid‐attenuated inversion recovery; Gd, gadolinium; GLCM, gray level co‐occurrence matrix; GLDM, gray level dependence matrix; GLRLM, grey‐level run length matrix; GLSZM, gray level size zone matrix; LASSO, least absolute shrinkage and selection operator; LSTM, long short‐term memory; MET‐PET, C‐methionine positron emission tomography; MGMT, O‐6‐Methylguanine‐DNA Methyltransferase; MTT, mean transit time; NGTDM, neighbourhood gray‐tone difference matrix; PsP, pseudoprogression; RFE, recursive feature elimination; RN, radiation necrosis; SUV, standardized uptake value; SVM, support vector machine; TTP, true tumor progression.

**Table A2:** Checklist for artificial intelligence in medical imaging (CLAIM)

Title/abstract	Gao 2020b	Liu 2020	Akbari 2020	Gao 2020a	Lohmann 2020	Lee 2020	Kebir 2020	Li 2020	Kim 2019	Elshafeey 2019	Bani‐Sadr 2019	Bacchi 2019	Jang 2018	Ismail 2018	Booth 2017	Zhang 2016	Artzi 2016	Prasanna 2016	Qian 2016	Hu 2011	Jang 2020	Peng 2018	Wang 2020	Zhang 2019	Zhang 2018	Total Score by Quality Indicator
Identification as a study of AI methodology, specifying the category of technology used (e.g., deep learning)	0	1	0	1	0	1	0	1	0	0	0	1	0	0	0	1	1	0	0	1	0	0	0	0	0	8
Structured summary of study design, methods, results, and conclusions	1	1	1	1	1	1	1	0	1	0	1	1	0	1	1	1	1	0	0	1	0	1	1	1	1	19
Introduction																										
Scientific and clinical background, including the intended use and clinical role of the AI approach	1	1	1	1	1	1	1	1	1	1	1	0	1	1	0	1	1	0	1	1	0	0	1	1	1	20
Study objectives and hypotheses	1	0	1	1	1	1	1	1	1	1	1	1	1	1	1	0	0	0	0	0	1	1	1	1	0	18
Methods																										
Study Design																										
Prospective or retrospective study	1	0	1	1	1	1	1	0	1	1	1	1	1	1	1	0	1	1	1	0	0	0	1	1	1	19
Study goal, such as model creation, exploratory study, feasibility study, non‐inferiority trial	1	1	1	1	1	1	1	1	1	1	1	1	1	1	1	0	1	1	1	0	1	1	1	1	1	23
Data																										
Data sources	1	1	1	1	1	1	1	1	1	1	1	1	1	1	0	1	0	1	1	1	1	1	1	0	0	21
Eligibility criteria: how, where, and when potentially eligible participants or studies were identified (e.g., symptoms, results from previous tests, inclusion in registry, patient‐care setting, location, dates)	1	0	1	1	0	0	1	0	1	1	1	0	1	1	1	0	1	1	0	0	1	1	1	1	0	16
Data pre‐processing steps	1	1	1	1	1	0	0	0	1	0	0	1	1	1	1	1	1	1	1	1	0	0	1	1	1	18
Selection of data subsets, if applicable	1	1	0	0	0	0	1	0	0	0	0	0	0	0	0	0	0	0	0	0	0	0	0	0	0	3
Definitions of data elements, with references to Common Data Elements	1	1	0	0	0	1	1	1	1	0	0	0	0	0	1	0	0	1	1	1	0	1	1	1	1	14
De‐identification methods	0	0	0	0	0	0	0	0	0	0	0	0	0	0	0	0	0	0	0	0	0	0	0	0	0	0
How missing data were handled	0	0	0	0	0	0	0	0	0	0	0	0	0	0	0	0	0	0	0	0	0	0	0	0	0	0
Ground truth																										
Definition of ground truth reference standard, in sufficient detail to allow replication	1	0	1	1	1	1	1	0	1	1	1	1	1	0	1	0	0	1	0	1	1	1	0	1	1	18
Rationale for choosing the reference standard (if alternatives exist)	0	0	1	1	0	1	0	0	0	1	0	0	0	0	0	0	1	0	0	0	1	1	0	0	0	7
Source of ground‐truth annotations; qualifications and preparation of annotators	0	0	1	1	0	0	0	0	1	1	1	0	0	0	0	0	1	0	0	0	0	1	0	1	0	8
Annotation tools	1	1	1	1	1	1	1	1	1	1	1	0	1	1	1	0	1	1	1	0	1	1	0	0	1	20
Measurement of inter‐ and intrarater variability; methods to mitigate variability and/or resolve discrepancies	1	0	1	0	0	0	0	0	0	0	0	0	0	0	1	0	0	0	0	0	0	0	1	0	0	4
Data partitions																										
Intended sample size and how it was determined	0	0	0	0	0	0	0	0	0	0	0	0	0	0	0	0	0	0	0	0	0	0	0	0	0	0
How data were assigned to partitions; specify proportions	1	1	1	1	1	0	1	1	1	1	1	0	1	0	0	0	0	0	0	0	0	0	1	0	0	12
Level at which partitions are disjoint (e.g., image, study, patient, institution)	1	1	1	1	1	0	1	0	1	1	1	1	1	1	1	0	0	1	0	1	0	0	1	0	0	16
Model																										
Detailed description of model, including inputs, outputs, all intermediate layers and connections	0	1	1	1	0	1	0	1	0	0	0	0	1	0	0	0	0	0	0	0	1	0	0	0	0	7
Software libraries, frameworks, and packages	1	1	1	1	1	1	1	0	1	1	1	0	1	0	1	1	1	0	1	0	0	1	1	1	1	19
Initialization of model parameters (e.g., randomization, transfer learning)	0	0	0	0	0	1	0	0	0	0	0	0	0	0	0	0	0	0	0	0	0	0	0	0	0	1
Training																										
Details of training approach, including data augmentation, hyperparameters, number of models trained	0	1	0	1	0	1	0	1	0	0	0	1	0	0	0	0	0	0	0	0	1	1	0	1	0	8
Method of selecting the final model	1	1	1	1	0	1	0	1	1	1	1	0	1	1	0	0	0	0	1	0	1	0	0	0	1	14
Ensembling techniques, if applicable	0	1	0	0	0	0	0	0	0	0	0	0	0	0	0	0	0	0	0	0	0	0	0	0	0	1
Evaluation																										
Metrics of model performance	1	1	1	1	0	1	1	1	1	1	1	1	1	0	1	1	1	1	1	1	1	1	1	1	1	23
Statistical measures of significance and uncertainty (e.g., confidence intervals)	0	0	0	1	0	0	1	0	1	0	1	0	0	0	1	0	0	0	1	0	0	0	1	0	0	7
Robustness or sensitivity analysis	1	1	1	1	1	1	1	1	1	1	1	0	1	0	1	0	0	1	1	0	0	1	0	1	0	17
Methods for explainability or interpretability (e.g., saliency maps), and how they were validated	1	1	1	1	0	1	1	1	0	1	1	0	1	0	0	1	0	0	1	0	1	1	1	0	1	16
Validation or testing on external data	0	0	1	0	0	0	0	0	1	0	0	0	0	1	0	0	0	0	0	0	1	0	0	0	0	4
Results																										
Data																										
Flow of participants or cases, using a diagram to indicate inclusion and exclusion	0	0	0	1	0	0	0	0	1	0	0	0	0	0	1	0	0	0	0	0	0	0	1	0	0	4
Demographic and clinical characteristics of cases in each partition	1	0	0	1	1	1	1	0	1	1	1	0	1	0	1	0	1	1	0	0	1	0	1	1	1	16
Model performance																										
Performance metrics for optimal model(s) on all data partitions	1	0	1	1	1	1	0	0	1	1	1	0	1	0	1		1	1	0	1	0	0	1	1	0	15
Estimates of diagnostic accuracy and their precision (such as 95% confidence intervals)	1	0	0	1	0	1	1	0	1	0	1	0	0	0	1	0	0	0	1	0	0	0	1	1	0	10
Failure analysis of incorrectly classified cases	0	0	0	0	0	0	0	0	0	0	0	0	0	0	0	0	0	0	0	0	0	0	0	0	0	0
Discussion																										
Study limitations, including potential bias, statistical uncertainty, and generalizability	1	1	1	1	1	1	1	0	1	1	1	1	1	1	1	0	0	1	0	1	1	1	1	1	1	21
Implications for practice, including the intended use and/or clinical role	1	0	1	1	1	0	0	0	1	1	1	0	1	0	1	1	1	0	1	1	1	1	1	0	0	16
Other information																										
Registration number and name of registry	0	0	0	0	0	0	0	0	0	0	0	0	0	0	0	0	0	0	0	0	0	0	0	0	0	0
Where the full study protocol can be accessed	0	0	0	0	0	0	0	0	0	0	0	0	0	0	0	0	0	0	0	0	0	0	0	0	0	0
Sources of funding and other support; role of funders	1	0	1	1	1	1	1	0	1	1	1	1	1	1	1	1	0	1	1	1	1	1	1	1	1	22
Total score by study	26	20	26	30	18	24	22	14	27	22	24	13	22	14	22	10	15	15	16	13	17	18	23	19	15	
